# Defect-Engineered VO_2_ Films: From Abrupt Phase Transition to Continuous Infrared Modulation via High-Vacuum Annealing

**DOI:** 10.3390/nano16100575

**Published:** 2026-05-08

**Authors:** Lin Liu, Jinxiao Li, Lei Wu, Xiaoling Wu, Guoan Cheng, Ruiting Zheng

**Affiliations:** School of Physics and Astronomy, Beijing Normal University, Beijing 100875, China; 201831220007@mail.bnu.edu.cn (L.L.); 202131220008@mail.bnu.edu.cn (J.L.); 202231220005@mail.bnu.edu.cn (L.W.); wuxl@bnu.edu.cn (X.W.)

**Keywords:** VO_2_ films, high-vacuum annealing, metal–insulator transition, defect engineering, infrared emissivity

## Abstract

Vanadium dioxide (VO_2_) films have attracted extensive attention for their pronounced metal–insulator transition (MIT) and multifunctional responses, holding great promise for smart windows, infrared stealth, memristive devices, and advanced sensors. However, conventional approaches for tuning the transition temperature, such as elemental doping or heterostructure engineering, often suffer from complicated processing, impurity phases, and poor device uniformity. Here, we use a dopant-free, high-vacuum annealing (9 × 10^−4^ Pa, ≈9 × 10^−6^ mbar) strategy to regulate the intrinsic structural evolution of VO_2_ films via oxygen-vacancy engineering and to clarify its influence on electrical switching contrast and infrared emissivity modulation. As the annealing temperature increases under low oxygen partial pressure, oxygen vacancies gradually accumulate, converting V^4+^ to V^3+^ and driving the films through three distinct structural stages: low-temperature lattice expansion with preserved M1 framework, critical structural collapse at 550 °C, and high-temperature defect rearrangement with local recrystallization. Consequently, the electrical MIT temperature continuously decreases, but the switching ratio collapses at the critical point and only partially recovers after high-temperature reorganization, while the infrared emissivity response transitions from abrupt, phase-transition-dominated switching to a continuous, tunable modulation at elevated temperatures. Notably, the infrared response begins continuous tuning earlier (≈450 °C) than the collapse of electrical MIT, reflecting the different sensitivities of optical and electronic responses to local lattice defects. These results reveal the coupling among oxygen-vacancy evolution, structural stability, electrical contrast, and infrared modulation in compositionally simple VO_2_ films. Compared with conventional doping, this high-vacuum annealing strategy avoids impurity phases, preserves compositional simplicity, and provides a scalable defect-engineering route to design VO_2_-based devices with reconfigurable electrical and infrared response modes.

## 1. Introduction

Vanadium dioxide (VO_2_) is a typical strongly correlated oxide that undergoes a reversible metal–insulator transition (MIT) near 68 °C, accompanied by a structural transformation from the monoclinic M1 phase to the rutile R phase and a dramatic electronic band reconstruction [1,2,3,4,5]. During this transition, the electrical conductivity can change by 4–5 orders of magnitude, while pronounced infrared and optical modulation effects are simultaneously observed [1,6,7,8]. In addition to thermal triggering, the MIT in VO_2_ can also be induced by optical excitation, electric field, and mechanical stress, making it a versatile platform for constructing multifunctional systems with multi-stimulus triggering and multimode responses [4,9,10,11].

Owing to these unique properties, VO_2_ thin films have been widely considered for applications in smart windows, infrared stealth, memristive devices, terahertz modulators, and sensors [8,12,13,14]. Nevertheless, the practical implementation of VO_2_-based devices still faces several major challenges. First, the intrinsic transition temperature (Tc) is relatively high for many applications. Second, although elemental doping and heterostructure engineering can reduce Tc, these methods often introduce impurity phases, degrade stability, and complicate large-area fabrication [15,16,17,18]. Third, lattice defects and interfacial effects are often difficult to control precisely, which severely limits the engineering tunability of the coupled electrical, optical, and structural properties [19,20,21].

Considerable efforts have been made to reduce the transition temperature of VO_2_, including high-valence doping, oxygen-vacancy engineering, strain/interface control, and composite or plasmonic structures [22,23,24,25]. W doping is one of the most effective approaches and can strongly lower Tc [22], while oxygen-vacancy modulation can also reduce Tc and the insulating-state resistance [23]. In addition, strain/interface engineering and metal/VO_2_ composite structures can tune the transition behavior through lattice distortion, interfacial stress, charge transfer, or percolation effects [24,25]. However, excessive dopants or defects often broaden the transition and reduce the electrical or optical contrast, while composite and interface designs may introduce additional coupled factors. Therefore, beyond lowering Tc, it is also important to clarify how intrinsic structural evolution affects the contrast and response mode of VO_2_ films.

In recent years, research interest has gradually shifted from single-parameter Tc modulation to the synergistic regulation of defects and multiscale structures. Several studies have pointed out that defect engineering, including oxygen vacancies, dislocation regions, and metastable phase conversion, provides an effective route to tailor phase-transition behavior without introducing foreign dopants [23,26]. For example, Sim et al. systematically demonstrated that oxygen vacancies, created by interfacial oxygen transport from VO_2_ to an epitaxial TiO_2_ layer, can dramatically lower the MIT temperature and reduce the insulating-state resistance [23]. A recent review by Valakh et al. summarizes that oxygen non-stoichiometry alone can lower the MIT temperature from about 340 K to as low as 142 K in bulk VO_2_ [26]. Furthermore, metastable phases such as the B phase and M2 phase can be stabilized through strain or self-doping effects without any external dopant [27,28,29,30]. These findings collectively highlight that intrinsic defects, such as oxygen vacancies, strain-induced dislocations, and metastable polymorph conversions, offer a powerful, dopant-free platform for continuously tuning the coupled electronic and structural phase transition in VO_2_.

From the perspective of application, previous studies have explored the influence of defects on the MIT and infrared properties of VO_2_ through methods such as magnetron sputtering, ion irradiation, and external-field stimulation [8,12,14,31,32,33]. However, systematic studies on the deoxygenation-induced structural evolution and property modulation of VO_2_ thin films under high-vacuum annealing remain limited [21,23,26]. In particular, the intrinsic relationship among oxygen-vacancy accumulation, structural reconstruction, electrical transition behavior, and infrared emissivity response has not yet been fully established.

In this work, we employ high-vacuum annealing as a dopant-free post-treatment method to regulate oxygen-defect levels and the intrinsic structural evolution of M1-phase VO_2_ films. By combining multiscale structural characterizations (SEM, Raman, XRD, XPS, and HRTEM) with electrical and infrared measurements, we investigate the continuous structural evolution of the films and their impact on the electrical MIT and infrared emissivity response. A three-stage defect–structure–property model is established, revealing how oxygen-vacancy accumulation first lowers the MIT temperature, then triggers structural collapse and suppresses abrupt switching, and finally drives defect rearrangement to convert the infrared response from an abrupt phase-transition-dominated mode to a gradual, continuous modulation mode. This transition from binary on/off behavior to continuously tunable infrared functionality is particularly promising for smart thermal management, infrared stealth, and neuromorphic computing applications. Overall, this study provides new insights into the coupling among oxygen-vacancy evolution, structural stability, electrical switching contrast, and infrared emissivity modulation in compositionally simple VO_2_ films [34], offering a scalable route for designing reconfigurable VO_2_-based response modes.

## 2. Experimental Methods

### 2.1. Sample Preparation

VO_2_ thin films were deposited on *c*-plane Al_2_O_3_ substrates by radio-frequency (RF) magnetron sputtering using a metallic V target with a purity of 99.9%. Prior to deposition, the substrates were ultrasonically cleaned in acetone, absolute ethanol, and deionized water for 20 min each, followed by drying with high-purity nitrogen gas. The base pressure of the sputtering chamber was evacuated to 1.2 × 10^−4^ Pa before deposition.

During deposition, the RF power was set to 150 W. The Ar and O_2_ flow rates were 49.1 and 0.96 sccm, respectively, corresponding to a working pressure of 0.5 Pa. The substrate temperature was maintained at 650 °C, the target-to-substrate distance was 15 cm, and the deposition time was 60 min. The thickness of the as-deposited films was approximately 80 nm.

Post-deposition annealing was performed in a vacuum chamber equipped with a microwave heating system. The samples were placed on a graphite holder, which offers excellent high-temperature stability and thermal conductivity, thus improving temperature uniformity during annealing. Before heating, the chamber pressure was reduced to 9 × 10^−4^ Pa (≈9 × 10^−6^ mbar), which was used as the high-vacuum annealing condition in this work. The samples were heated to the target temperature within approximately 15 min and then held for 2 h, followed by natural cooling to room temperature in vacuum. The annealing temperatures were 350, 400, 450, 550, 650, 750, and 850 °C, and the corresponding samples were labeled as VO_2_-350 °C, VO_2_-400 °C, VO_2_-450 °C, VO_2_-550 °C, VO_2_-650 °C, VO_2_-750 °C, and VO_2_-850 °C, respectively.

### 2.2. Characterization

The surface morphology of the films was characterized by field-emission scanning electron microscopy (SEM, S-4800, Hitachi High-Tech Corporation, Tokyo, Japan) at an accelerating voltage of 10 kV and a working distance of 8.0 mm. To improve conductivity during observation, the samples were coated with a thin Pt layer when necessary.

Raman spectra were collected using a micro-Raman spectrometer (Renishaw in Via Reflex spectrometer Renishaw plc, Wotton-under-Edge, Gloucestershire, UK) with a 532 nm excitation laser. The laser power was set to 5 mW, with an exposure time of 10 s and three accumulations for each spectrum.

Phase composition and crystal structure were analyzed by X-ray diffraction (XRD, PANalytical X’Pert PRO MPD, Malvern Panalytical B.V., Almelo, The Netherlands) using Cu Kα radiation (λ = 0.15418 nm) over a 2θ range of 10–90° in the conventional θ–2θ mode.

Local microstructure was further examined by scanning transmission electron microscopy (STEM, FEI Tecnai G2 F20, Thermo Fisher Scientific, Hillsboro, OR, USA) operated at 200 kV. Cross-sectional TEM samples were prepared by focused ion beam (FIB) milling. High-resolution imaging, selected-area electron diffraction, and Fourier analysis were used to examine local structural characteristics.

X-ray photoelectron spectroscopy (XPS, ESCALAB250Xi, Thermo Fisher Scientific, Waltham, MA, USA) was used to analyze elemental composition and chemical valence states. Before data acquisition, the sample surfaces were mildly cleaned by Ar^+^ sputtering for 30 s to remove adventitious carbon and surface adsorbates. The binding energies were calibrated using the C1s peak at 284.8 eV. The O1s and full V2p regions were fitted using a Shirley-type background and mixed Gaussian–Lorentzian peak functions. For the V2p spectra, the spin–orbit doublets of V^3+^ and V^4+^ were included, with the V2p_3/2_:V2p_1/2_ area ratio fixed at 2:1 and the spin–orbit splitting constrained within 7.3–7.6 eV [35]. The same constrained fitting strategy was applied to all samples.

Electrical properties were measured using a four-probe testing system consisting of a Keithley DMM6500 digital multimeter (Keithley Instruments, LLC, Cleveland, OH, USA), a semiconductor heating stage, a temperature-control module, four-point electrodes, a purge gas system, and a water-cooling unit. The measurement temperature range was from −10 to 100 °C. Resistance and temperature were recorded simultaneously during heating and cooling at a rate of approximately 0.2 °C s^−1^, with 1280 data points collected in each measurement cycle.

Infrared response was measured using a custom-built temperature-controlled infrared characterization system consisting of a semiconductor heating stage, a temperature controller, a circulating water-cooling system, and an infrared camera (Optotherm IS640, Optotherm Inc., Marlborough, MA, USA) equipped with a macro lens. The maximum spatial resolution was 140 μm, the working distance was approximately 10 cm, and the measurement temperature range was 36–100 °C. Infrared images were acquired and analyzed using Optotherm Thermalyze software 7.6.0.6.

## 3. Results and Discussion

### 3.1. Morphological and Structural Evolution of VO_2_ Films

**Figure 1:**
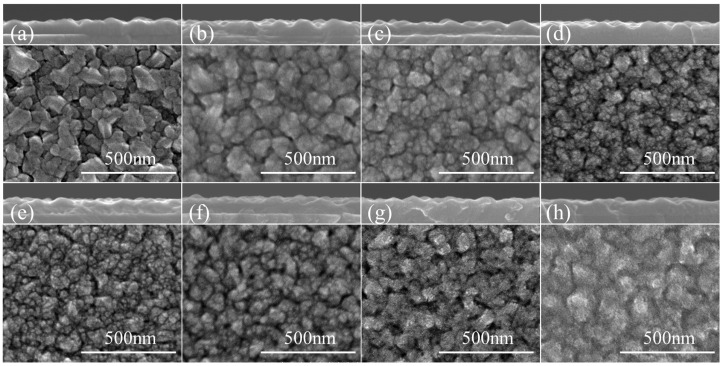
SEM images of VO_2_ films after high-vacuum annealing at different temperatures. (**a**) Pristine film, (**b**–**h**) Samples annealed at 350, 400, 450, 550, 650, 750, and 850 °C, respectively.

Figure 1 shows the SEM images of the as-deposited VO_2_ film (a) and the films annealed at 350 °C (b), 400 °C (c), 450 °C (d), 550 °C (e), 650 °C (f), 750 °C (g), and 850 °C (h). The as-deposited film exhibits a dense surface with well-defined grains and a relatively uniform size distribution, with only a few abnormally large grains; the average grain size is ~78 nm. The cross-sectional image indicates good continuity and integrity of the film, with a thickness of ~69 nm. After annealing at 350 °C, the granular morphology is largely preserved, whereas the grain edges become less faceted and more rounded; the average grain size slightly decreases to ~69 nm and the film thickness remains nearly unchanged. Further increasing the annealing temperature to 400 and 450 °C, the fraction of fine grains increases while the initially larger grains are progressively fragmented/refined, reducing the average apparent grain size to ~54 nm and ~42 nm, respectively; meanwhile, the surface becomes more compact and exhibits a clear tendency toward fragmentation, although grain boundaries remain identifiable.

When the annealing temperature reaches 550 °C, a more dramatic morphological transition occurs. The grains are further refined to approximately 30–45 nm, accompanied by clustering, fragmentation, and local discontinuities. This indicates that the VO_2_ film is no longer in a regime of simple defect perturbation but has entered a structurally unstable state. Upon further increasing the annealing temperature to 650 °C and above, the grain size shows a tendency to increase again, while local aggregation, pores, and blurred boundaries appear. These features imply enhanced atomic mobility at elevated temperatures, which promotes thermally driven reorganization and recrystallization. Overall, the grain size follows a nonmonotonic trend, decreasing first and then increasing, with a minimum near 550 °C, providing morphological evidence for a critical structural transition temperature.

**Figure 2:**
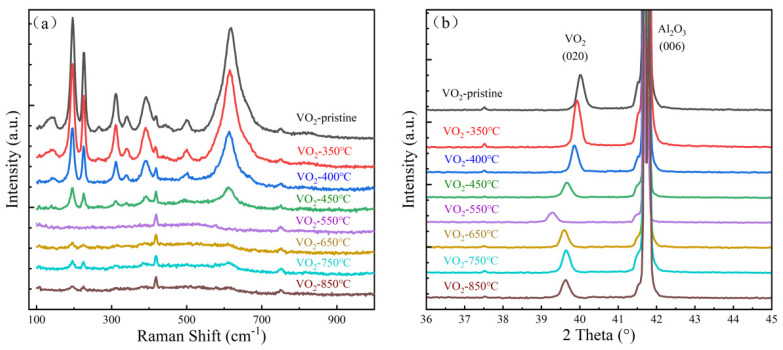
(**a**) Raman spectra and (**b**) XRD patterns of VO_2_ films after high-vacuum annealing at different temperatures.

To further clarify the phase and crystal-structure evolution induced by vacuum annealing, Raman spectra and XRD patterns of the films are shown in Figure 2a,b. In the as-deposited sample and the samples annealed at 350–450 °C, the Raman spectra exhibit the characteristic vibrational modes of monoclinic VO_2_ (M1) near 197, 225, 390, and 615 cm^−1^, indicating that the film remains dominated by the M1 phase in this temperature range [6,32,36]. Therefore, the main effect in this stage is defect accumulation and lattice perturbation rather than a complete phase transition.

At 550 °C, the characteristic Raman peaks of the M1 phase almost disappear, indicating that the original monoclinic framework has been severely disrupted and the film has entered a new structural state. The XRD results also reveal significant deviations from the low-temperature samples, confirming that 550 °C is a key threshold for structural reconstruction in VO_2_ thin films under high-vacuum annealing. When the annealing temperature is further increased to 650 °C and above, some Raman features associated with the M1 phase reappear, suggesting that recrystallization occurs at high temperatures and that some local regions recover M1-like structural motifs, although their defect background, local stress state, and lattice environment differ substantially from those of the pristine film. This order–disorder–reordering tendency is broadly consistent with the known polymorphic instability and phase reconstruction behavior of vanadium dioxide under thermal treatment [27,29,30].

The XRD patterns further reveal the continuous evolution of the average lattice parameters. As the annealing temperature increases from 350 to 550 °C, the VO_2_ (020) diffraction peak gradually shifts toward lower angles, indicating an increase in the interplanar spacing and thus expansion along the *b*-axis. This result suggests that high-vacuum annealing significantly modifies the average lattice state of the film and intensifies lattice perturbation within this temperature range. Notably, when the annealing temperature exceeds 550 °C, the (020) peak begins to shift back toward higher angles and eventually tends to stabilize, implying that the structural evolution at high temperature is no longer a simple monotonic lattice expansion but instead involves local structural rearrangement, stress relaxation, and recrystallization.

Taken together, the SEM, Raman, and XRD results allow the structural evolution of VO_2_ thin films under high-vacuum annealing to be divided into three representative stages: (i) Low-temperature structural perturbation stage (350–450 °C): the films remain M1-dominated, while grain refinement and progressive (020) peak shift indicate lattice expansion and increasing structural disturbance; (ii) Critical structural instability stage (550 °C): M1 Raman features nearly disappear, the surface becomes highly fragmented, and XRD features become anomalous, indicating pronounced structural instability and reconstruction; (iii) High-temperature structural reorganization stage (≥650 °C): M1 structural characteristics are reconstructed through defect migration, aggregation, and rearrangement, while the film does not return to the pristine low-defect state but evolves into a structurally reorganized state with redistributed defects.

### 3.2. Oxygen Vacancies and V-Valence Evolution of VO_2_ Films

After establishing the three-stage structural evolution, the key issue is to identify its chemical origin. Since high-vacuum annealing at 9 × 10^−4^ Pa (≈9 × 10^−6^ mbar) directly lowers the oxygen chemical potential around the film, it is expected to affect lattice oxygen stability and the local valence state of V ions. Therefore, XPS measurements were performed on representative samples annealed at 400, 550, and 850 °C, corresponding to the low-temperature perturbation stage, the critical structural-instability stage, and the high-temperature reorganization stage, respectively.

**Figure 3:**
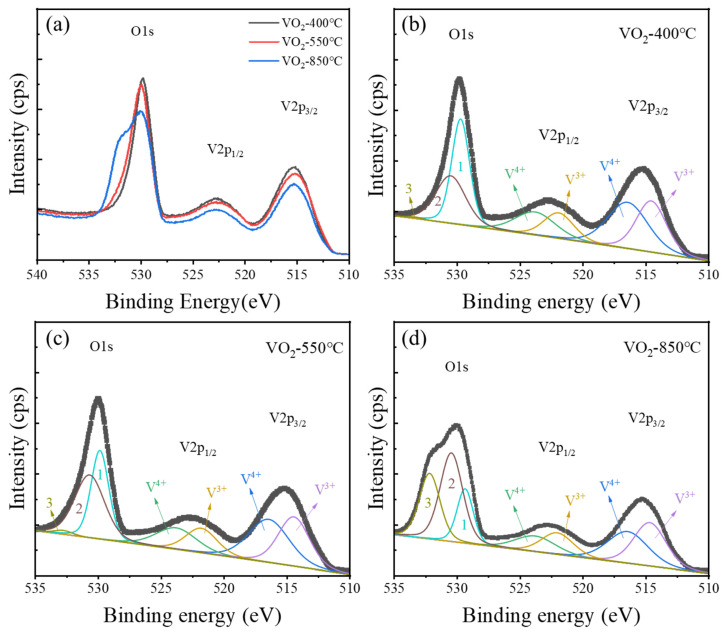
XPS analysis of VO_2_ films after high-vacuum annealing. (**a**) Survey spectra of the films annealed at different temperatures; (**b**–**d**) fitted O1s and V2p spectra of the films annealed at 400, 550, and 850 °C, respectively. The O1s components labeled 1, 2, and 3 correspond to lattice oxygen, oxygen-vacancy-related/nonideal oxygen environments, and adsorbed oxygen or surface species, respectively.

Figure 3a presents the XPS survey spectra, while Figure 3b–d show the fitted O1s and full V2p regions based on constrained V^3+^/V^4+^ spin–orbit doublets. The O1s spectra were decomposed into three components: a lattice oxygen component at approximately 530.0 eV, an oxygen-vacancy-related/nonideal oxygen component near 531.2 eV, and a higher-binding-energy component related to adsorbed oxygen or surface species. As the annealing temperature increases from 400 to 850 °C, the relative contribution of the oxygen-vacancy-related component increases and the O1s peak becomes broader, supporting the formation and accumulation of oxygen vacancies during high-vacuum annealing.

The fitting results of the V valence states are summarized in Table 1. The full V2p spectra were fitted using constrained V^3+^ and V^4+^ spin–orbit doublets, and the films mainly contain V^4+^ and V^3+^ species. With increasing annealing temperature, the proportion of V^3+^ gradually increases from 44.94% in the 400 °C sample to 46.80% in the 550 °C sample and 52.00% in the 850 °C sample, while the V^4+^ fraction decreases correspondingly. This result demonstrates that the films undergo a progressive reduction process under high-temperature and high-vacuum conditions, and that the average vanadium valence shifts toward lower oxidation states. Since oxygen release from the lattice must be accompanied by local charge compensation, the conversion of V^4+^ to V^3+^ occurs cooperatively with the increase in oxygen-vacancy concentration. Similar defect-induced valence evolution has been reported previously in vanadium oxide thin films and related VO_2_ systems [21,35,37]. In essence, this reflects a progressive weakening of the V–O bond, deterioration of the local coordination environment, and continuous reduction in lattice stability during annealing.

Importantly, the significance of the XPS results is not merely that “higher temperature leads to more oxygen vacancies,” but rather that they provide the chemical origin of the structural evolution observed above. In the low-temperature stage, represented by the 400 °C sample, a moderate concentration of oxygen vacancies introduces local lattice distortion and changes the carrier concentration, yet is insufficient to destroy the M1 framework; therefore, the system exhibits a lowered MIT threshold while retaining structural integrity. At 550 °C, the further increase in oxygen vacancies and V^3+^ concentration substantially weakens the local V-O octahedral coordination environment, while the cooperative V-V pairing and lattice correlations that stabilize the M1 phase are disrupted, leading to structural instability and the emergence of a new structural state. In the high-temperature reorganization stage, represented by the 850 °C sample, the increased V^3+^ fraction indicates that the oxygen-vacancy concentration does not decrease but instead continues to increase. However, the system no longer evolves simply toward increased disorder; rather, it enters a regime dominated by defect migration, aggregation, and rearrangement, through which a new local structural balance is established under thermal diffusion.

### 3.3. Local Microstructure of VO_2_ Films

To further verify whether the films indeed undergo a local evolution from order to enhanced disorder and then to partial reordering, HRTEM analysis was carried out on the pristine sample, the 550 °C sample, and the 850 °C sample. The latter two were selected because they represent the most significant structurally unstable state and the final high-temperature reorganized state, respectively.

**Figure 4:**
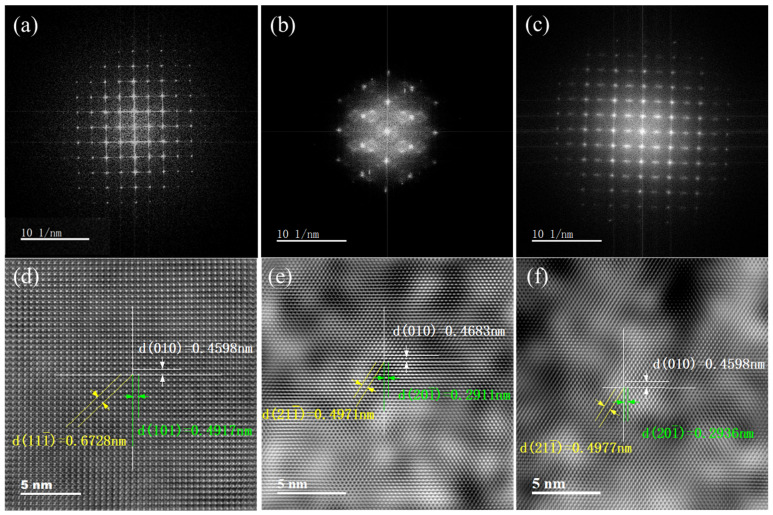
Microstructural evolution of VO_2_ thin films under different annealing temperatures. (**a**–**c**) Fast Fourier transform (FFT) patterns of HRTEM images of the annealed samples: (**a**) pristine, (**b**) 550 °C, and (**c**) 850 °C; (**d**–**f**) Inverse Fourier transform (IFFT) filtered lattice images of the corresponding regions: (**d**) Pristine film, (**e**) VO_2_-550 °C, (**f**) VO_2_-850 °C.

Figure 4a–c show the fast Fourier transform (FFT) patterns obtained from the HRTEM images of these samples. The pristine VO_2_ film exhibits clear, regular, and well-defined diffraction spots, indicating high local crystallinity and good structural ordering. After annealing at 550 °C, the FFT pattern shows a marked reduction in the number of diffraction spots, accompanied by pronounced diffuse scattering, demonstrating a substantial decrease in local lattice order. Combined with the XPS results, this confirms that the large increase in oxygen-vacancy concentration weakens the V-O coordination environment, enhances lattice distortion, and ultimately drives local structural reconstruction. When the annealing temperature is increased further to 850 °C, more regular and denser diffraction spots reappear in the FFT pattern, indicating that the local degree of order increases again. However, the diffraction features differ from those of the pristine sample, implying that high-temperature annealing does not simply restore the original structure, but instead produces a new local equilibrium state after defect rearrangement and structural reorganization. This behavior is in line with prior reports showing that VO_2_-related metastable structures and strained states can undergo irreversible reconstruction into new equilibria rather than fully revert to the initial lattice [27,28,29,30].

Figure 4d–f show the inverse fast Fourier transform (IFFT) filtered HRTEM images of the corresponding regions. In the pristine film, clear and continuous lattice fringes can be identified, with interplanar spacings of d(010) = 0.4598 nm, d(101) = 0.4917 nm, and d(11$\bar{1}$) = 0.6728 nm, indicating a relatively intact lattice arrangement. After annealing at 550 °C, the measured spacings become d(010) = 0.4683 nm, d(20$\bar{1}$) = 0.2911 nm, and d(21$\bar{1}$) = 0.4971 nm. The enlarged d(010) spacing indicates lattice expansion or enhanced local distortion along this direction, consistent with the low-angle shift in the (020) peak in XRD. This agreement strongly supports the conclusion that oxygen-vacancy accumulation in the critical stage leads to local structural relaxation and internal stress redistribution. After annealing at 850 °C, d(010) returns to approximately 0.4598 nm, while the other lattice spacings and fringe configurations still differ from those of the pristine sample. This indicates that high-temperature treatment partially restores certain lattice parameters but does not recover the original structure; instead, a new locally ordered state is formed through defect rearrangement and structural reconstruction.

It should be emphasized that XRD and HRTEM provide complementary structural information at different length scales. XRD reflects the average lattice parameters over a relatively large volume, so the back-shift in the (020) peak and the reduction in average interplanar spacing at temperatures above 550 °C mainly indicate global structural reorganization and stress relaxation, rather than a reduction in oxygen-vacancy concentration. In contrast, the increased V^3+^ content observed by XPS indicates that the defect level remains high in the high-temperature samples. HRTEM further confirms that, at elevated temperatures, oxygen vacancies do not disappear; instead, they migrate, aggregate, and rearrange, thereby promoting local reordering in some regions while maintaining a strong defect background in others. In other words, the high-temperature samples are characterized by an average structure that appears relatively stabilized, but a local defect structure that has been substantially reorganized.

### 3.4. Influence of Structural Evolution on Electrical MIT and Infrared Emissivity Response

**Figure 5:**
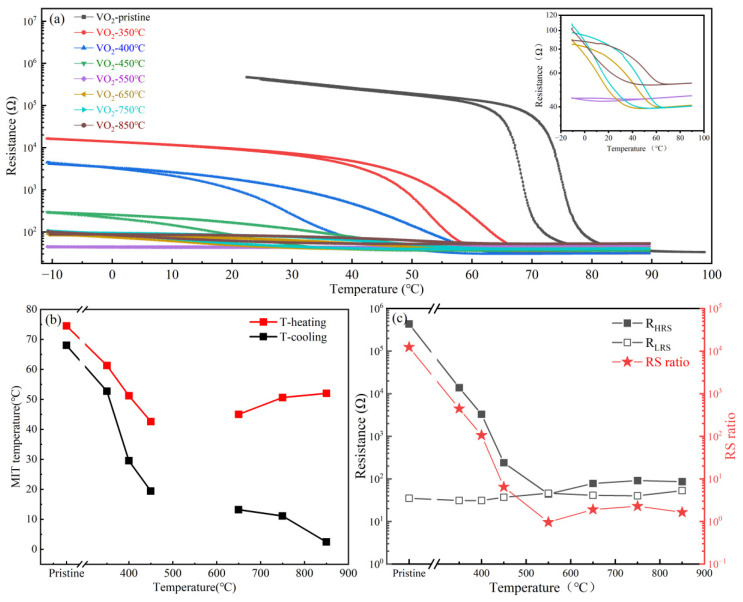
Electrical response testing of VO_2_ films after high-vacuum annealing at different temperatures. (**a**) Variation in film resistance with temperature. (inset) An enlarged view of the corresponding resistance-temperature curve. (**b**) Variation in the film phase transition temperature with annealing temperature. (**c**) Variation in high-/low-temperature resistance and electrical switching ratio with annealing temperature.

**Figure 6:**
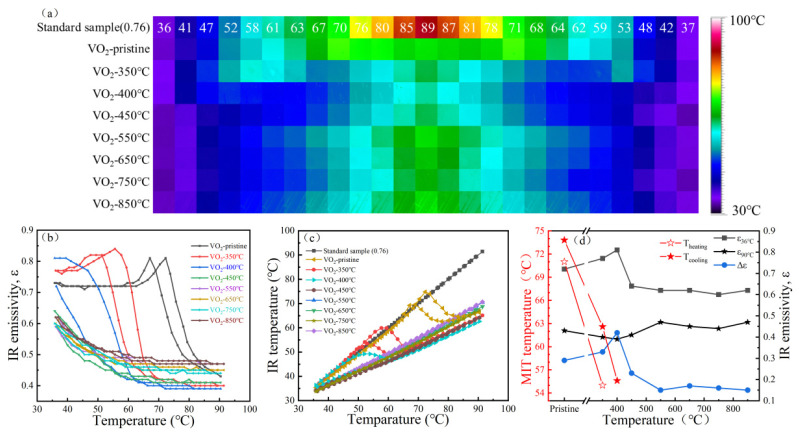
Infrared response testing of VO_2_ films after high-vacuum annealing at different temperatures. (**a**) Color-scale map showing the infrared detection temperature of the film during heating and cooling cycles, (**b**) Variation in the film’s infrared emissivity with environmental temperature, (**c**) Relationship between the film’s infrared detection temperature and environmental temperature, (**d**) Variation in the film’s infrared response performance with annealing temperature.

After clarifying the defect evolution and structural reconstruction caused by high-vacuum annealing, it is essential to understand how these structural changes are reflected in the functional responses of the VO_2_ films. For practical VO_2_-based devices, the key issue is not only the shift in transition temperature, but also the retention, loss, or transformation of electrical and infrared contrast during defect-driven structural evolution. Figure 5 and Figure 6 summarize the resistance–temperature behavior and infrared response of the films after different annealing treatments.

#### 3.4.1. Low-Temperature Structural Perturbation Stage

As shown in Figure 5a, the pristine VO_2_ film exhibits a typical and sharp MIT behavior, with a resistance change spanning approximately four orders of magnitude, indicating excellent reversible phase-transition characteristics [1,4,5]. After annealing at 350–450 °C, the films still retain obvious resistance jumps, but the transition temperature is significantly reduced, reaching as low as about 51.2 °C, while the electrical switching ratio remains above 10^2^. This electrical response is attributed to moderate oxygen-vacancy accumulation, which increases free carrier concentration and induces local lattice distortion, thereby lowering the phase-transition barrier while preserving the M1 framework. Thus, the low-temperature annealing stage mainly shifts the transition threshold while maintaining a measurable resistance contrast.

The infrared response in this stage is highly consistent with the electrical behavior, as shown in Figure 6. The pristine sample exhibits a typical abrupt infrared emissivity modulation during heating and cooling, with an infrared emissivity modulation amplitude (Δε) of approximately 0.30, corresponding to the rapid change in optical constants during the MIT [6,8,38]. The infrared response remains abrupt, with Δε ≈ 0.42–0.44 for treatments below 400 °C. This indicates that low-temperature defect regulation lowers the MIT temperature while improving the infrared modulation contrast of VO_2_. In this stage, oxygen vacancies modulate the optical constants (n, k) without disrupting the cooperative M1 ↔ R transition.

Therefore, in the low-temperature stage, the primary role of oxygen vacancies is to tune the phase-transition threshold, rather than to alter the fundamental phase-transition mechanism. The dominant response remains the complete M1 ↔ R transition, but its critical temperature is shifted downward.

#### 3.4.2. Critical Structural Instability Stage

At 550 °C, the resistance–temperature curve shows almost no pronounced transition, indicating near-complete suppression of the MIT. This is due to severe destruction of the M1-dominated structural framework, combined with oxygen-vacancy accumulation and lattice distortion, which eliminates the structural basis for a reversible insulating-to-metallic transition and collapses the electrical resistance contrast to near unity.

Similarly, the infrared response evolves from the abrupt behavior of the pristine film to a flat, continuous profile, reflecting a progressive reduction in Δε. Notably, this transition toward a continuous infrared response begins as early as 450 °C, with Δε ≈ 0.23, preceding the complete suppression of the electrical MIT observed at 550 °C. This asynchronous behavior arises because the infrared emissivity is governed by macroscopic optical constants (n, k) averaged over the film, so it can respond to local structural inhomogeneity differently and earlier than the percolative electrical MIT. At 550 °C, the infrared modulation drops to its lowest level (Δε ≈ 0.1), and the response curve becomes flatter, corresponding to the near disappearance of the electrical MIT. This indicates that the structural reconstruction in the critical window fundamentally alters the infrared response mechanism: the film no longer maintains a complete and cooperative first-order M1 ↔ R transition pathway, and the optical constants consequently lose the structural basis for a sharp emissivity jump.

Thus, 550 °C is not merely the point of “worst performance,” but the most important mechanistic turning point in this work: at this stage, the system switches from a phase-transition-dominated response to a structural-reconstruction-dominated response.

#### 3.4.3. High-Temperature Structural Reorganization Stage

For the samples annealed at 650 °C and above, part of the MIT behavior reappears in the resistance curves, but the transition amplitude is much smaller than that of the pristine sample, and both the high- and low-temperature resistance values are altered. This indicates that, although the high-temperature reorganized films locally reform structural units with M1-like characteristics, the system still retains a high concentration of oxygen vacancies and structural inhomogeneity. Consequently, the phase-transition behavior is not equivalent to that of the original VO_2_ film, but only partially restored. In other words, high-temperature annealing does not return the samples to their initial state, but leads instead to a new defect–structure equilibrium, in which local regions may still undergo phase transition while the whole film can no longer exhibit the original sharp and collective MIT.

The infrared results (Figure 6) further support this conclusion. For the samples annealed at 650–850 °C, the infrared response remains continuous and smooth (Δε ≈ 0.15–0.17), reflecting the averaged optical effect of multiple local domains under a high-defect background rather than sharp, cooperative transitions. This transformation from an abrupt switching mode to a continuous tuning mode is closely related to the high-defect, polycrystalline, and partially recrystallized microstructure formed after high-temperature reorganization. Because the material no longer behaves as a single and complete M1 ↔ R phase-transition system, but instead consists of multiple local structural regions under a high-defect background, its macroscopic infrared response becomes continuous rather than sharply switching near a specific critical temperature.

Overall, the evolution of electrical and infrared responses can be understood as two facets of the same oxygen-vacancy-driven structural evolution process. In the low-temperature stage, moderate defect concentrations lower the MIT threshold while preserving the M1 framework, producing abrupt electrical and infrared responses. In the critical stage, structural instability collapses the cooperative MIT pathway, causing disappearance of abrupt transitions in both measurements, albeit at different temperatures. In the high-temperature stage, defect rearrangement and local recrystallization establish a new structural equilibrium, partially restoring electrical MIT in local domains while infrared response transitions to a continuous modulation mode. These observations demonstrate that high-vacuum annealing progressively shifts VO_2_ from a phase-transition-dominated regime under low-defect conditions to a structural-reconstruction-dominated regime under high-defect conditions, emphasizing the oxygen-vacancy–structure–performance coupling as the key factor governing the asynchronous electrical and infrared responses and the transformation of functional contrast.

## 4. Conclusions

In summary, we demonstrate that high-vacuum annealing enables a dopant-free, thermally driven route to continuously tune the electrical and infrared responses of VO_2_ thin films through intrinsic oxygen-vacancy evolution and structural regulation. A three-stage defect–structure–property evolution is identified. At low temperatures (350–450 °C), moderate oxygen vacancies lower the MIT temperature while preserving M1 framework, resulting in abrupt electrical and infrared responses. At the critical window (≈550 °C), severe lattice distortion suppresses MIT and reduces electrical switching, while infrared response transitions to a smooth, continuous profile due to macroscopic optical constant averaging. At high temperatures (≥650 °C), grain coarsening and defect redistribution allow partial MIT recovery, yet the infrared modulation remains continuous. These results reveal how transition-temperature tuning, electrical resistance contrast, and infrared emissivity modulation are jointly governed by oxygen-vacancy-driven structural evolution. This transition from a binary on/off phase-change behavior to a continuously tunable infrared response is particularly attractive for smart thermal management, infrared stealth, and neuromorphic computing applications. Compared with conventional elemental doping, our high-vacuum annealing strategy avoids impurity phases, maintains compositional simplicity, and offers a scalable path for engineering VO_2_-based multifunctional devices with reconfigurable response modes.

## Figures and Tables

**Table 1:** Fitting results of the V valence states in VO_2_ films annealed at 400, 550, and 850 °C. The V2p_3/2_:V2p_1/2_ area ratio was fixed at 2:1, and the spin–orbit splitting was constrained within 7.3–7.6 eV.

	V^4+^2p_3/2_ BE/eV	V^4+^2p_1/2_ BE/eV	ΔE (V^4+^) eV	V^4+^/%	V^3+^2p_3/2_ BE/eV	V^3+^2p_1/2_ BE/eV	ΔE (V^3+^) eV	V^3+^/%
400 °C	516.45	523.80	7.35	55.06	514.58	521.93	7.35	44.94
550 °C	516.45	523.80	7.35	53.20	514.46	521.81	7.35	46.80
850 °C	516.45	523.80	7.35	48.00	514.69	522.04	7.35	52.00

## Data Availability

The original contributions presented in this study are included in the article. Further inquiries can be directed to the corresponding authors.
